# Efficiency estimates for electromicrobial production of branched-chain hydrocarbons

**DOI:** 10.1016/j.isci.2023.108773

**Published:** 2023-12-21

**Authors:** Timothy J. Sheppard, David A. Specht, Buz Barstow

**Affiliations:** 1 Department of Biological and Environmental Engineering, Cornell University, Ithaca, NY 14853, USA

**Keywords:** Chemistry, Electrochemistry, Electrochemical materials science, Interfacial electrochemistry

## Abstract

In electromicrobial production (EMP), electricity is used as microbial energy to produce complex molecules starting from simple compounds like CO_2_. The aviation industry requires sustainable fuel alternatives that can meet demands for high-altitude performance and modern emissions standards. EMP of jet fuel components provides a unique opportunity to generate fuel blends compatible with modern engines producing net-neutral emissions. Branched-chain hydrocarbons modulate the boiling and freezing points of liquid fuels at high altitudes. In this study, we analyze the pathways necessary to generate branched-chain hydrocarbons *in vivo* utilizing extracellular electron uptake (EEU) and H_2_-oxidation for electron delivery, the Calvin cycle for CO_2_-fixation and the aldehyde deformolating oxygenase decarboxylation pathway. We find the maximum electrical-to-fuel energy conversion efficiencies to be $40.0_{-4.4}^{+0.6}\%$ and $39.8_{-4.5}^{+0.7}\%$. For a model blend containing straight-chain, branched-chain, and terpenoid components, increasing the fraction of branched-chain alkanes from zero to 47% only lowers the electrical energy conversion efficiency from $40.1_{-4.5}^{+0.7}\%$ to $39.5_{-4.6}^{+0.7}\%$.

## Highlights

• Electromicrobial production could support the aviation sector’s need for biofuels
• A relevant fuel blend could be produced at efficiencies higher than convention
• A combination of differentially structured hydrocarbons can achieve drop-in fuels
• Branched-chain hydrocarbons can be made at efficiencies similar to straight-chains

## Graphical abstract

**Figure:**
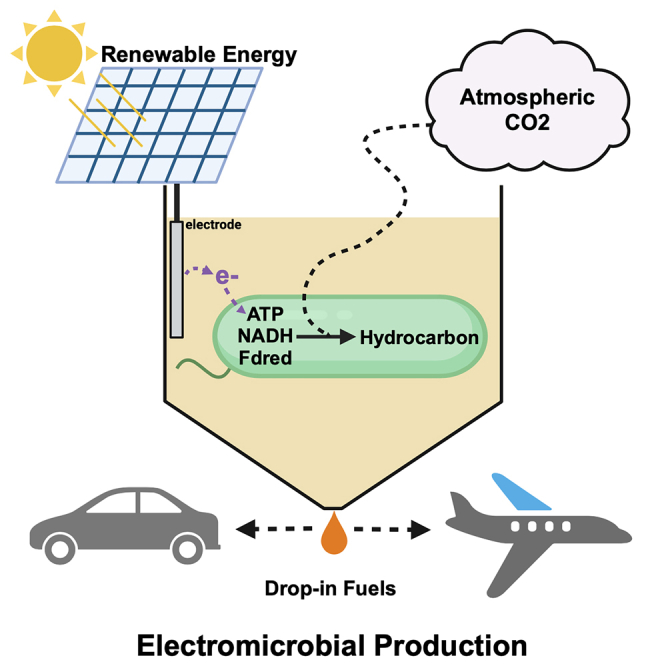

## Introduction

The industrial-scale synthesis of carbon-neutral hydrocarbon fuels that are drop-in compatible with present-day internal combustion and jet engines is one of the biggest challenges in the decarbonization of the world’s energy infrastructure. Corn ethanol can be safely blended into gasoline up to a fraction of ≈10–15%. However, the widespread use of high fractions of ethanol in fuel blends is unfeasible as it lacks the high boiling and low freezing points needed in many applications, especially aviation.^1^ This difficulty has motivated the production of kerosene-grade biofuel blends. Third-generation algal biofuels show promise for use in aviation, but the upscaling of algal fuels is challenging and faces many hurdles to commercial acceptance.^2^ For alternative fuels to be established as commonplace, their properties must be as close to those of conventional fuels as possible.

In addition to straight-chain alkanes and terpenoids, branched-chain hydrocarbons are key components of traditional jet fuels. In Jet A-1, branched-chain hydrocarbons are used to raise the boiling and lower the freezing points while burning almost as cleanly as straight-chain alkanes.^3^ Although terpenoids (whose electromicrobial synthesis was described by us previously^4^) could achieve similar boiling increases and freezing point reductions, they also create significant soot deposition during combustion.^5^ Production of a library of branched-chain hydrocarbons could permit synthesis of blends that closely match the composition and physico-chemical properties of fossil-derived kerosene, while also burning cleanly. Furthermore, gasoline containing a high fraction of isoalkanes (one type of branched-chain alkanes) could burn much cleaner than conventional gasoline.^6^

Electromicrobial production (EMP) could enable highly efficient production of carbon-neutral drop-in biofuels. EMP is a broadly encompassing term for a group of technologies that aim to combine electricity and microbial metabolism for conversion of simple compounds into complex, energy dense molecules like food and biofuels.^7^^,^^8^^,^^9^^,^^10^^,^^11^^,^^12^^,^^13^^,^^14^^,^^15^^,^^16^ EMP has allowed for microbes that assimilate electrochemically reduced CO_2_ like formate^14^^,^^17^ and acetate;^16^ H_2_-oxidizing, CO_2_-fixing systems like the Bionic Leaf;^18^^,^^19^ microbe-semiconductor hybrids;^20^ and microbes that can directly absorb electricity through processes like extracellular electron uptake (EEU).^8^^,^^21^^,^^22^ Lab-scale demonstrations of EMP already have effective solar-to-chemical energy conversion efficiencies exceeding all forms of terrestrial photosynthesis.^19^^,^^23^ Meanwhile, theoretical predictions indicate that the efficiency of EMP could exceed all forms of photosynthesis.^7^^,^^8^^,^^24^^,^^25^^,^^26^ This high efficiency mitigates many of the concerns about competition for land created by first- and second-generation biofuels.^27^^,^^28^ Furthermore, a large library of metabolic pathways for the biological synthesis of branched-chain hydrocarbons has been established^3^^,^^6^^,^^29^^,^^30^^,^^31^^,^^32^ that could allow the production of jet fuel blends much closer in composition to Jet A-1 than algae-derived biofuels (reviewed in Adesina et al.^33^ and Sheppard et al.^34^).

In this work we extend our earlier predictions of EMP efficiency^8^^,^^25^^,^^26^^,^^34^ to make minimum energy cost and upper-limit production efficiency estimates of single- and multi-branched hydrocarbons powered by H_2_-oxidation^18^^,^^19^ or EEU,^8^^,^^21^^,^^22^ with carbon supplied by *in vivo* CO_2_-fixation with the Calvin cycle. We then calculate the production efficiency of drop-in fuel blends of increasing branched-chain content.

## Results

### Electromicrobial production of jet fuel components

We predict upper limit efficiencies for the EMP of branched-chain hydrocarbons. These predictions set an upper bound on the performance of a set of highly engineered microorganisms created for production of drop-in jet fuel components. Below we summarize all of the key equations utilized in this article. For detailed derivations see Salimijazi et al.^8^ and our subsequent work that builds upon this theory.^25^^,^^26^^,^^34^ All model parameters are shown in Table 1, and all symbols used in this article are shown in Table S1.

**Table 1:** Electromicrobial jet fuel production model parameters

Parameter	Symbol	1. H_2_	2. EEU
***Electrochemical Cell Parameters***			

Input solar power (W)	*P*_*γ*_	1,000	1,000
Total available electrical power (W)	*P*_e, total_	330	330
CO_2_-fixation method		Enzymatic	
Electrode to microbe mediator		H_2_	EEU
Cell 2 (Bio-cell) anode std. potential (V)	*U*_cell 2, anode, 0_	−0.41 (ref.^18^)	−0.1 (ref.^35^ and ^36^)
Bio-cell anode bias voltage (V)	*U*_cell 2, anode, bias_	0.3 (ref.^19^)	0.2 (ref.^37^)
Bio-cell cathode std. potential (V)	*U*_cell 2, cathode, 0_	0.82	
Bio-cell cathode bias voltage (V)	*U*_cell 2, cathode, bias_	0.47	
Bio-cell voltage (V)	Δ*U*_cell 2_	2 (ref.^19^)	1.59
Bio-cell Faradaic efficiency	*ξ*_I2_	1.0	

***Cellular Electron Transport Parameters***			

Membrane potential difference (mV)	Δ*U*_membrane_	140	
Terminal *e*^−^ acceptor potential (V)	*U*_Acceptor_	0.82	
Quinone potential (V)	*U*_Q_	−0.0885 (ref.^35^)	
Mtr EET complex potential (V)	*U*_Mtr_	N/A	−0.1 (ref.^8^)
No. protons pumped per *e*^-^	*p*_out_	Unlimited	

***Product Synthesis Parameters***			

No. ATPs for product synthesis	*ν*_p, ATP_	See Table S2	
No. NAD(P)H for product	*ν*_p, NADH_	See Table S2	
No. Fd_red_ for product	*ν*_p, Fd_	See Table S2	
Product energy density (J molecule^−1^)	*E*_HC_	See Table S3	

Model parameters used in this article are based upon model parameters used in a previous analysis of the electromicrobial production of the biofuel butanol.^8^ A sensitivity analysis was performed for all key parameters in this work.^8^

**Figure 1:**
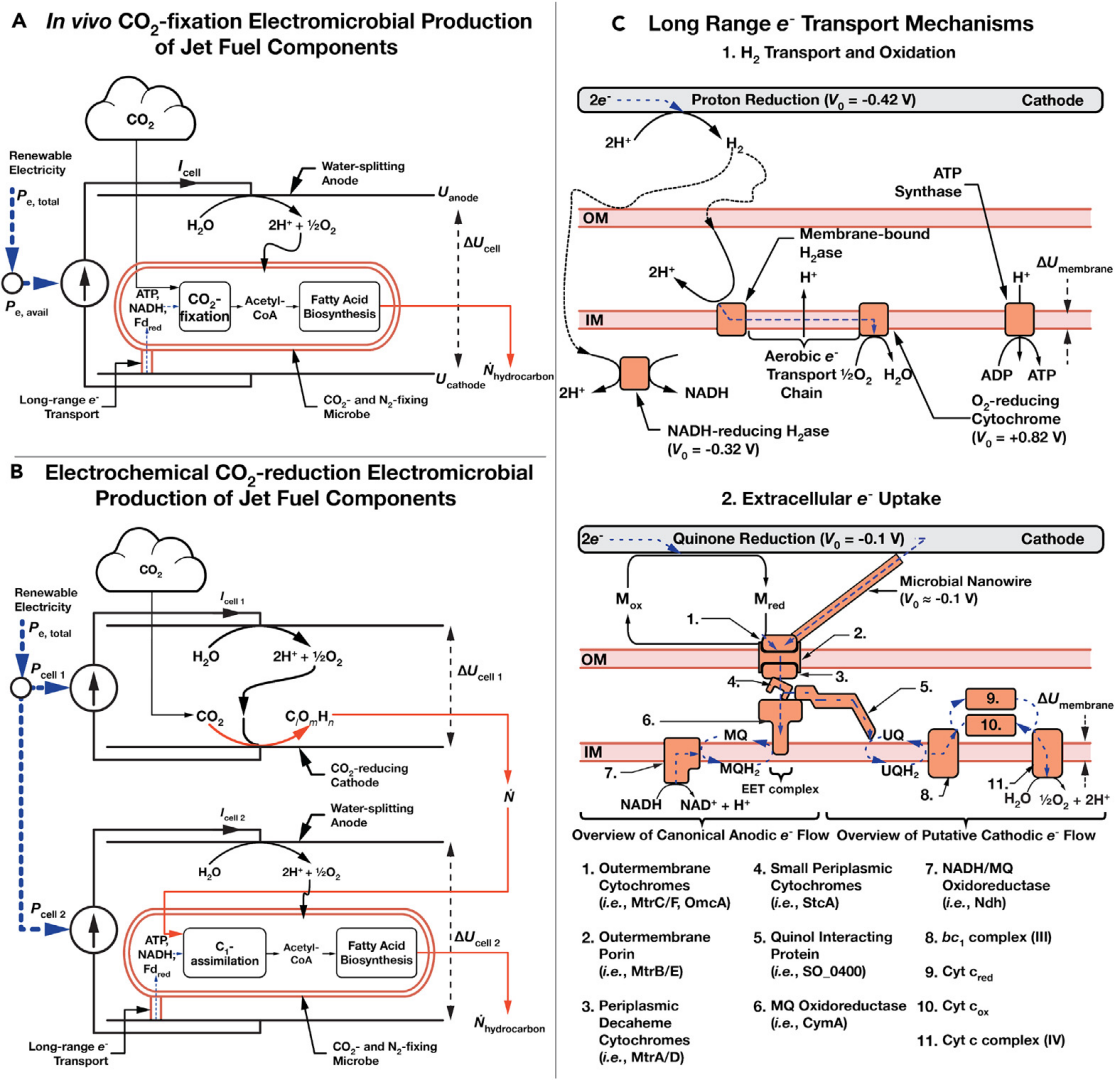
Schematic of electromicrobial production of jet fuel components (A) In the article we just consider electromicrobial production systems that use the Calvin-Benson-Bassham (CBB) cycle for *in vivo* CO_2_-fixation and hydrocarbon synthesis. (B) Mechanism by which electricity sources can be used to power microbial production, using either H_2_-oxidation or extracellular electron uptake (EEU). (C) In the first, H_2_ is electrochemically reduced on a cathode, transferred to the microbe by diffusion or stirring, and is enzymatically oxidized. In the second mechanism, extracellular electron uptake (EEU), electrons are transferred from a cathode (*i*) along a microbial nanowire (part of a conductive biofilm), (*ii*) by a reduced medium potential redox shuttle like a quinone or flavin, or (*iii*, not shown) by direct contact of the cell with the cathode and are then oxidized at the cell surface by the extracellular electron transfer (EET) complex. From the thermodynamic perspective considered in this article, these mechanisms of EEU are equivalent. Electrons are then transported to the inner membrane where reverse electron transport is used to regenerate NAD(P)H, reduced Ferredoxin (not shown), and ATP.^8^^,^^21^^,^^39^ This schematic is modified from our earlier work on the synthesis of the straight-chain alkane and terpenoid components of jet fuels.^34^

As in earlier work, we assume access to a reservoir of CO_2_. Reducing power for the regeneration of NAD(P)H and ATP are provided via oxidation of electrochemically reduced H_2_ (Figure 1B part 1) by EEU from a diffusible intermediary (such as flavins or anthra(hydra)quinone-2,6-disulfonate (AHDS_red_/AQDS_ox_)) or through a conductive biofilm or direct contact with a cathode (Figure 1B part 2). We assume that the energy requirements for microbial maintenance are negligible at maximum efficiency, allowing the cell to operate as a “bag of enzymes”.^8^^,^^38^

Hydrocarbon molecules with an energy-per-molecule, *E*_HC_, are produced a rate of *Ṅ*_HC_ molecules per second. The amount of energy needed to produce a mole of hydrocarbon, *L*_EP_,^8^^,^^25^

(Equation 1) $$L_{\text{EP}}=P_{e,T}N_{A}/{\overset{\cdot }{N}}_{\text{HC}}\text{,}$$

where $P_{e,T}$ is the power input to the system and *N*_A_ is the Avogadro constant. Thus, the minimum energy input into the bio-electrochemical system is,

(Equation 2) $$L_{\text{EP}}\geq N_{A}\Delta U_{e.\text{cell}}e\nu_{\text{ep}}\text{,}$$

where Δ*U*_e.cell_ is the potential difference across the bio-electrochemical cell (note we have changed this from Δ*U*_cell_ in earlier work for clarity), *e* is the fundamental charge, and *ν*_ep_ is the number of electrons needed to synthesize a molecule of the product from CO_2_. The whole-cell voltage is one of the biggest determinants of EMP efficiency. In H_2_-oxidation systems, we make the assumption that the whole-cell voltage, Δ*U*_e.cell_, is 2V (the optimal applied voltage for the Bionic Leaf device^19^). However, it is not clear if this very low whole-cell voltage can be achieved in a scaled-up system, possibly due to mass transport issues. Increasing this value will reduce the efficiencies quoted here. For example, increasing Δ*U*_e.cell_ from 2V to 3V will reduce the efficiency by a factor of 2/3. A similar reduction will be seen for EEU-mediated systems.

Furthermore, the efficiency of energy conversion from input power to final product is,

(Equation 3) $$\eta_{\text{EP}}={\overset{\cdot }{N}}_{\text{HC}}E_{\text{HC}}/P_{e,T}\text{.}$$

When utilizing *in vivo* carbon-fixation (Figure 1A), the upper limit of electrical-to-chemical efficiency is equivalent to the energy carried per molecule of hydrocarbon, *E*_HC_, relative to the amount of energy needed to move the charge for product synthesis across the bio-electrochemical cell (*eν*_ep_Δ*U*_e.cell_),^8^

(Equation 4) $$\eta_{\text{EP}}\leq E_{\text{HC}}/\left(e\nu_{\text{ep}}\Delta U_{e.\text{cell}}\right)\text{.}$$

In this article we calculate the number of electrons needed for production of a hydrocarbon by *in vivo* CO_2_-fixation (*ν*_ep_) with electron uptake both by H_2_-oxidation and EEU.^8^ For electron delivery by H_2_,

(Equation 5) $$\nu_{\text{ep},H_{2}}=2\nu_{p,\text{NADH}}+2\nu_{p,\text{Fd}}+\nu_{p,\text{ATP}}\frac{\text{ceil}\left(\Delta G_{\text{ATP}/\text{ADP}}/e\Delta U_{\text{membrane}}\right)}{\text{floor}\left(\left(U_{H_{2}}-U_{\text{acceptor}}\right)/\Delta U_{\text{membrane}}\right)}\text{,}$$

where *ν*_p, NADH_, *ν*_p, Fd_, and *ν*_p, ATP_ are the number of NAD(P)H, reduced ferredoxin, and ATP needed for product synthesis; Δ*G*_ATP/ADP_ is the Gibbs free energy for regeneration of ATP; Δ*U*_membrane_ is the potential difference between the cytoplasmic and periplasmic faces of the inner membrane (the host of the electron transport chain); $U_{H_{2}}$ is the redox potential of H_2_-oxidation; and *U*_acceptor_ is the redox potential of the terminal electron acceptor (usually O_2_).

For electron delivery by EEU,

(Equation 6) $$\nu_{\text{ep},\text{EEU}}=2\nu_{p,\text{NADH}}+2\nu_{p,\text{Fd}}+\nu_{p,\text{ATP}}\frac{\text{ceil}\left(\Delta G_{\text{ATP}/\text{ADP}}/e\Delta U_{\text{membrane}}\right)}{\text{floor}\left(\left(U_{Q}-U_{\text{acceptor}}\right)/\Delta U_{\text{membrane}}\right)}+\nu_{p,\text{NADH}}\frac{\text{ceil}\left(\left(U_{\text{NADH}}-U_{Q}\right)/\Delta U_{\text{membrane}}\right)}{\text{floor}\left(\left(U_{Q}-U_{\text{acceptor}}\right)/\Delta U_{\text{membrane}}\right)}+\nu_{p,\text{Fd}}\frac{\text{ceil}\left(\left(U_{\text{Fd}}-U_{Q}\right)/\Delta U_{\text{membrane}}\right)}{\text{floor}\left(\left(U_{Q}-U_{\text{acceptor}}\right)/\Delta U_{\text{membrane}}\right)}\text{,}$$

where *U*_NADH_ is the redox potential of NAD(P)H reduction; *U*_Q_ is the redox potential of menaquinone reduction; and *U*_Fd_ is the redox potential of ferredoxin reduction.

### ATP, NAD(P)H, and reduced ferredoxin demands for jet fuel component electromicrobial production

The ATP, NADP(H), and reduced ferredoxin requirements for individual molecules in a jet fuel blend are calculated by flux balance analysis. Pathways for the production of the straight-chain alkane and terpenoid components of jet fuel were compiled by us in a recent article.^34^

**Figure 2:**
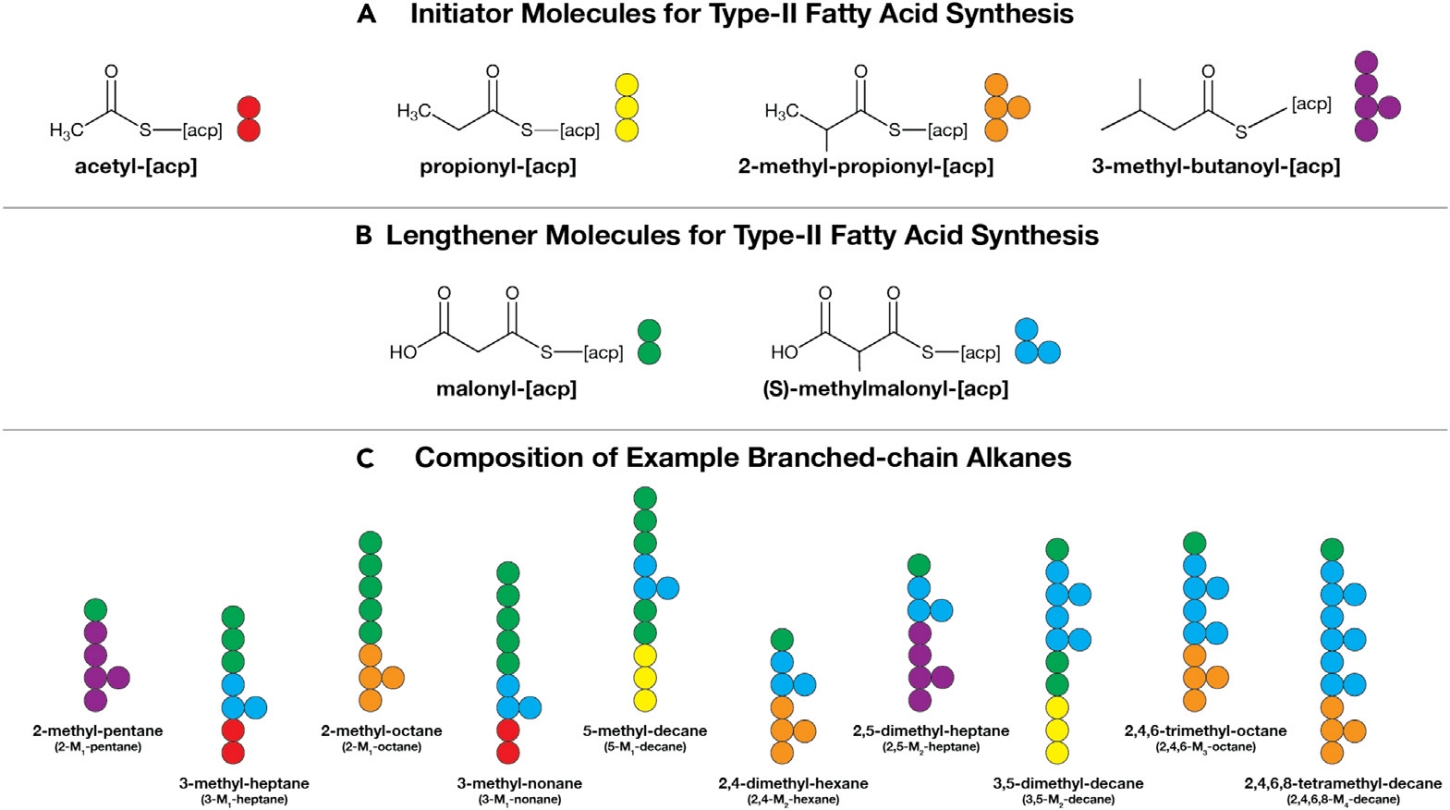
Mechanics for branched-chain alkane production Branched-chain alkanes are synthesized by the same Type II fatty acid synthase (FAS) system as straight-chain alkanes,^34^ but branches are added with additional initiator and lengthener molecules. (A) Initiators for branched-chain alkane synthesis. Acetyl-[acp] (acp: acyl-carrier protein) and propionyl-[acp] are also used as initiators for synthesis of even and odd chain-length straight-chain alkanes by Type II fatty acid synthase.^34^ 2-methyl-propionyl-[acp] and 3-methyl-butanoyl-[acp] are used exclusively for branched-chain alkanes by Type II fatty acid synthase. (B) Lengtheners for branched-chain alkane synthesis. Malonyl-[acp] is used to add two additional carbons to a growing straight- or branched-chain alkane. (S)-methylmalonyl-[acp] is used to add a branch to a growing branched-chain alkane. In all cases considered in this article, the last carbon in the alkane is by a termination reaction catalyzed by the well known aldehyde deformolating oxygenase (ADO) pathway. (C) Composition of example branched-chain alkane molecules shown in Figure 4. Synthesis pathways for initiators and lengtheners are shown in Figure 3. Pathways are shown in Table 2. Note that the position of the branch is normally measured from the bottom (the start of synthesis) of the molecule, but in the case of 3-M_1_-hexane and 5-M_1_-decane it is measured from the top of the chain.

**Figure 3:**
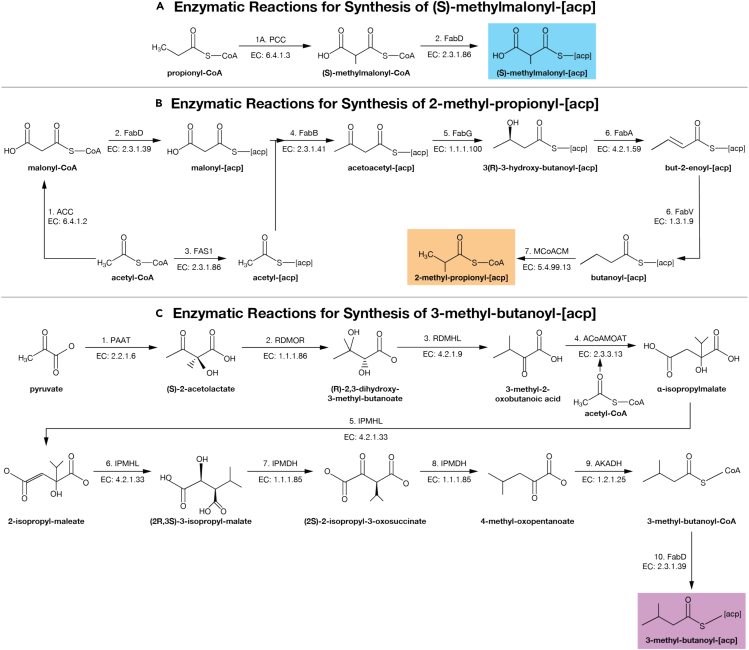
Enzymatic pathways for branched-chain component production Synthesis pathways for (A) the branched-chain lengthener (S)-methylmalonyl-[acp] and branched-chain initiators (B) 2-methyl-propionyl-[acp] and (C) 3-methyl-butanoyl-[acp]. Full pathways collected from Kyoto Encyclopedia of Genes and Genomes.^41^^,^^42^^,^^43^ (S)-methylmalonyl-[acp] pathway mediated by either Propionyl-CoA Carboxylase (PCC) or the downregulation of a naturally occurring regulatory enzyme Methylmalonyl-CoA Demethylase (MMCD) that traditionally prevents (S)-methylmalonyl-CoA formation from ACC (not depicted here). Full pathways are listed in Table 2.

**Figure 4:**
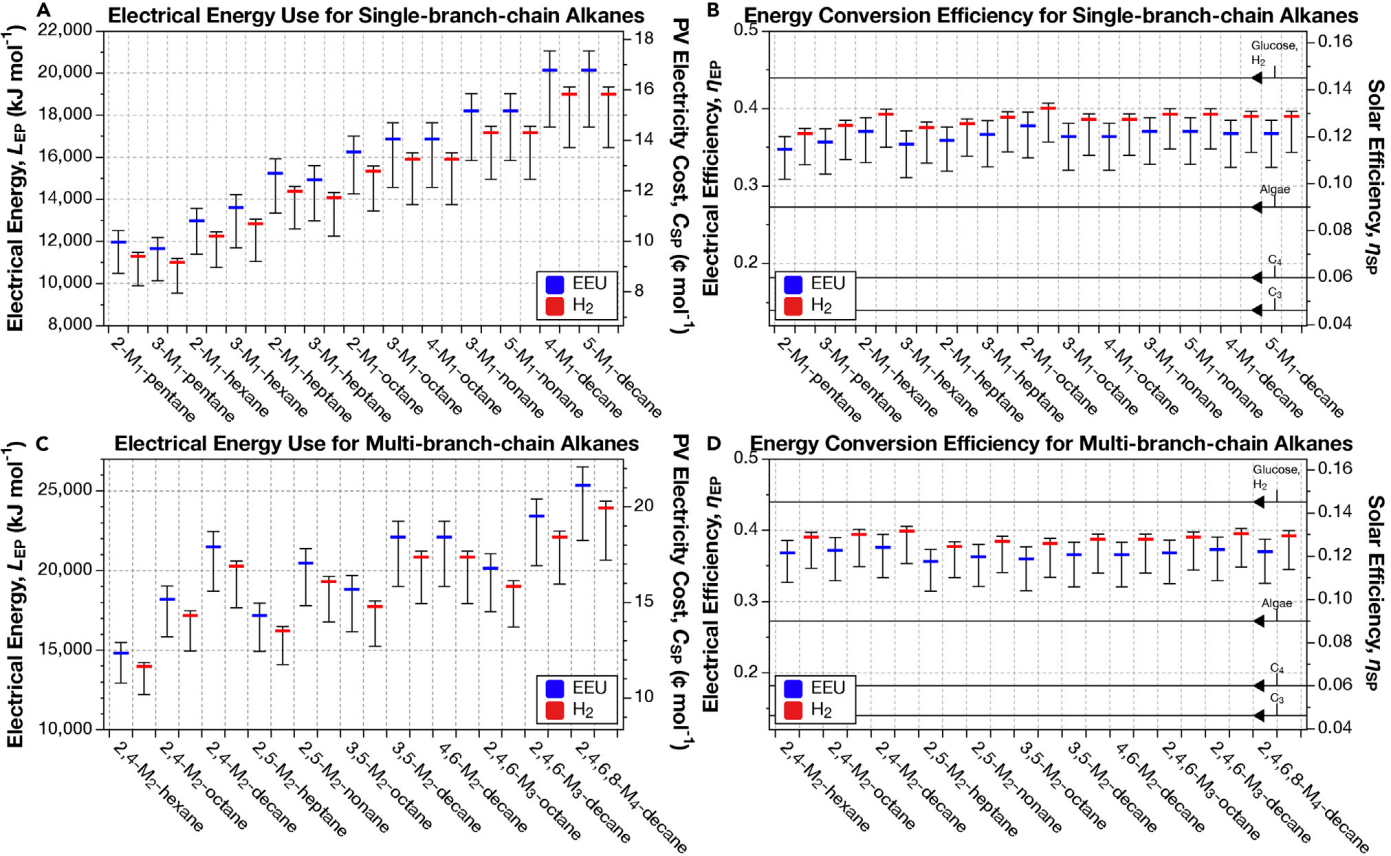
Electrical energy requirements and energy conversion efficiencies for single- and multi-branched-chain alkane production yields maximum efficiencies of 40.0% and 39.9%, respectively (A) Energy input for single-branched-chain fatty alkane biosynthesis using the Calvin CO_2_-fixation cycle with the ADO alkane termination pathway. (B) Energy conversion efficiency of single-branched-chain from solar cell on left axis. Solar conversion efficiency compared to C_3_, C_4_, algae, and H_2_-mediated electromicrobial production of glucose on right axis, lines corresponding to those in (D). (C) Energy input required for multi-branched-chain alkane biosynthesis using the Calvin CO_2_-fixation cycle with the ADO alkane termination pathway. (D) Energy conversion efficiency of multi-branched-chain alkane compound biosynthesis on left axis. Solar conversion efficiency compared to C_3_, C_4_, algae, and H_2_-mediated electromicrobial production of glucose on right axis, lines corresponding to those in (B). A sensitivity analysis by Salimijazi et al.^8^ found that the biggest source of uncertainty in the energy input and efficiency calculation is the potential difference across the inner membrane of the cell (Δ*U*_membrane_). Estimates for the *trans*-membrane voltage range from 80 mV (BioNumber ID^45^ (BNID) 10408284 to 270 mV (BNID 107135), with a most likely value of 140 mV (BNIDs 109774, 103386, and 109775). The central value (thick blue or red bar) corresponds to 140 mV. Our sensitivity analysis found that Δ*U*_membrane_ = 280 mV produces lower efficiencies (hence a higher energy input), while Δ*U*_membrane_ = 80 mV produces higher efficiencies (and hence lower energy inputs).^8^ The right axis in (A and C) shows the minimum cost of that solar electricity, assuming that the United States Department of Energy’s cost target of 3 ¢ per kWh by 2030 can be achieved.^46^ The right axes in (B and D) show the solar-to-product energy conversion efficiency, assuming the system is supplied by a perfectly efficient single-junction Si solar photovoltaic (solar to electrical efficiency of 32.9%.^47^ For comparison, we have marked the upper limit solar-to-biomass energy conversion efficiencies of C_3_, C_4_,^48^^,^^49^ algal photosynthesis,^50^ and upper limit electromicrobial production conversion efficiency of glucose using H_2_-oxidation and the Calvin cycle^25^ on the right axes of (B and D**)**. This figure can be reproduced by running the codes Info-Fig-4A&B.py, Info-Fig-4C&D.py, Fig-4A&B.py, and Fig-4C&D.py in the emp-to-branched-jet online code repository.^44^

Like straight-chain alkanes, branched-chain alkanes are produced by the Type II fatty acid synthase (FAS) system followed by decarboxylation.^3^ Branches are introduced into the growing fatty acid by incorporation of unconventional methylated initiator and lengthener molecules.^3^ An overview of branched-chain alkane synthesis is shown in Figure 2. Synthesis pathways for methylated initiators and lengtheners are shown in Figure 3 and Table 2. In wild-type cells, the incorporation of methylated initiators and lengtheners into fatty acids is kept at low levels and are limited by one of several regulatory enzymes native to these systems.^3^^,^^40^ Downregulation of these native regulatory enzymes can promote methylated initiator production and a high output of branched-chain hydrocarbons. From this start point, we are able to generate single-branched compounds of any length with odd or even methylation patterns as shown in Figure 3. Full pathways for the synthesis of a panel of individual branched-chain alkane compounds (shown in Figure 4) are compiled from listings of reactions in the Kyoto Encyclopedia of Genes and Genomes (KEGG)^41^^,^^42^^,^^43^ in the input files to the Info-Fig 4A&B.py, Info-Fig 4C&D.py codes in the emp-to-branched-jet repository.^44^

**Table 2:** Reactions for synthesis of initiator and lengthener molecules used for branched-chain alkane production

No.	Reaction	E.C. Number	KEGG Accession Code
	**(S)-methylmalonyl-[acp]**		
1A	ATP + propionyl-CoA + HCO_3_^−^ → ADP + orthophosphate + (S)-methylmalonyl-CoA	6.4.1.3	R01859
1B	ATP + propionyl-CoA + HCO_3_^−^ → ADP + orthophosphate + (S)-methylmalonyl-CoA	6.4.1.2	R00742
2	(S)-methylmalonyl-CoA + H-[acp] → (S)-methylmalonyl—[acp] + H-CoA	2.3.1.39	R01626
	**2-methyl-propionyl—[acp]**		
1	ATP + acetyl-CoA + HCO_3_^−^ → ADP + orthophosphate + malonyl-CoA	6.4.1.2	R00742
2	Malonyl-CoA + H-[acp] → malonyl-[acp] + H-CoA	2.3.1.39	R01626
3	Acetyl-CoA + H-[acp] → acetyl-[acp] + H-CoA	2.3.1.86	R01624
4	Acetyl-[acp] + malonyl-[acp] → acetoacetyl-[acp] + CO_2_ + CoA	2.3.1.41	R04355
5	Acetoacetyl-[acp] + NADPH + H^+^ → 3R-3-hydroxybutanoyl-[acp] + NADP^+^	1.1.1.100	R02767
6	3R-3-hydroxybutanoyl-[acp] → but-2-enoyl-[acp] + H_2_O	4.2.1.59	R04428
7	But-2-enoyl-[acp] + NADPH + H^+^ → butanoyl-[acp] + NADP^+^	1.3.1.9	R04429
8	Butanoyl-[acp] → 2-methyl-propanoyl-[acp]	5.4.99.3	R03052
	**3-methyl-butanoyl—[acp]**		
1	2 × pyruvate → (S)-2-acetolactate + CO_2_	2.2.1.6	R00006
2	(S)-2-acetolactate → 3-hydroxy-3-methyl-2-oxobutanoic acid	1.1.1.86	R04439
3	(R)-2,3-Dihydroxy-3-methylbutanoate → 3-methyl-2-oxobutanoic acid + H_2_O	4.2.1.9	R01209
4	Acetyl-CoA + 3-Methyl-2-oxobutanoic acid + H_2_O → α-isopropylmalate + CoA	2.3.3.13	R01213
5	α-isopropylmalate → 2-isopropylmalate + H_2_O	4.2.1.33	R03968
6	2-isopropylmalate + H_2_O → (2R,3S)-3-isopropylmalate	4.2.1.33	R04001
7	(2R,3S)-3-isopropylmalate + NAD^+^ → (2S)-2-isopropyl-3-oxosuccinate + NADH + H^+^	1.1.1.85	R01652
8	(2S)-2-isopropyl-3-oxosuccinate → 4-methyl-2-oxopentanoate + CO_2_	1.1.1.85	R01652
12	4-methyl-2-oxopentanoate + CoA + NAD^+^ → 3-methyl-butanoyl-CoA + CO_2_ + NADH + H^+^	1.2.1.25	R01651
12	3-methyl-butanoyl-CoA + H-[acp] → 3-methyl-butanoyl-[acp] + H-CoA	2.3.1.39	R01626

Full pathways collected from the Kyoto Encyclopedia of Genes and Genomes.^41^^,^^42^^,^^43^ The (S)-methylmalonyl-[acp] pathway is mediated by either Propionyl-CoA Carboxylase (PCC) or the downregulation of the regulatory enzyme Methylmalonyl-CoA Demethylase (MMCD) that traditionally prevents (S)-methylmalonyl-CoA formation from Acetyl-CoA Carboxylase. Pathways are depicted in Figure 3.

The overall stoichiometric matrix (**S**_**p**_) for synthesis of each alkane using the Calvin-Benson-Bassham cycle was calculated by the Info-Fig 4A&B.py and Info-Fig 4C&D.py codes.^44^ Briefly, we consider a chemical species number rate of change vector, ***ṅ***, that encodes the rate of change of number of the reactant molecules over a single cycle of the reaction network; a stoichiometric matrix **S**_p_ that encodes the number of reactants made or consumed in every reaction in the network; and a flux vector ***v*** that encodes the number of times each reaction is used in the network. Reactant molecules are denoted as inputs (e.g., CO_2_, ATP, NAD(P)H), outputs (e.g., H_2_O), intermediates, or the target molecule (e.g., the alkane to be synthesized). For the purposes of this thermodynamic analysis, we consider NADH and NADPH to be equivalent as they have near identical redox potentials. The number of NAD(P)H, reduced ferredoxin and ATP for each individual alkane are calculated by numerically solving the flux balance equation,

(Equation 7) $$\overset{\cdot }{n}=S_{p}v\text{,}$$

under the constraint that the number of each intermediate chemical species does not change over a reaction cycle, and that the number of target molecules increases by 1,

(Equation 8) $${\overset{\cdot }{n}}_{i}=\{\begin{array}{c} 0\;\text{if}\;\text{species}\;i\;\text{is}\;\text{an}\;\text{intermediate}\\ 1\;\text{if}\;\text{species}\;i\;\text{is}\;\text{an}\;\text{input}/\text{output}\;\text{or}\;\text{the}\;\text{target} \end{array}\text{.}$$

**Figure 5:**
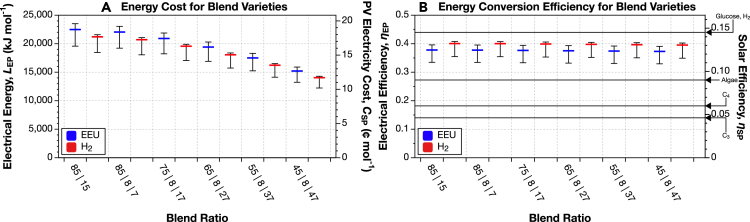
Adding branched-chain hydrocarbons to a fuel blend lowers production efficiencies by only ∼0.1% for every 10% branched-chains added Effect of increasing branched chain content on (A) energy input for, and (B) energy conversion efficiency of production of a model jet fuel blend containing equimolar proportions of straight-chains | terpenoids | and branched-chains. Right axes in (A) and (B) and lines in (B) correspond to those in Figure 4. The central value (thick blue or red bar) corresponds to the most likely value of the *trans*-membrane (Δ*U*_membrane_) voltage of 140 mV. Meanwhile, Δ*U*_membrane_ = 280 mV produces lower efficiencies (hence a higher energy input), while Δ*U*_membrane_ = 80 mV produces higher efficiencies (and hence lower energy inputs).^8^ This figure can be reproduced by running the codes Info-Fig-5A&B.py, and Fig-5A&B.py in the emp-to-branched-jet online code repository.^44^

The calculated stoichiometries for the synthesis of each branched-chain hydrocarbon considered in this article are listed in Table S2. The stoichiometries for each molecule are then combined with their molecular weights and energies per molecule (listed in Table S3) to calculate the energy input and production efficiency (Fig-4A&B.py; Fig-4A&B.py, results shown in Figure 4). The energy inputs and conversion efficiencies for jet fuel blends are calculated by a weighted average of the energy inputs and efficiencies of the individual components (Figure 5).

### Restrictions of branched-chain formation

Due to the limits of FAS, we are unable to produce adjacently methylated branched-chain alkanes. Given the molecular structure of malonyl-CoA, a methyl group can only be added to every second carbon during lengthening. This prevents the preparation of some single-methyl- and multi-methylated-branched-chains. Nonetheless, FAS allows us to produce a great variety of compounds that allow us to more closely mimic the composition of traditional jet fuel blends.

### Production of (S)-methylmalonyl-[acp] lengthener

Branches are added to a growing alkane during lengthening by incorporation of a (S)-methylmalonyl-[acp]. Natively, (S)-methylmalonyl-CoA is produced via Propionyl-CoA Carboxylase, which adds a single carbon to a propionyl-CoA molecule necessary for initiating branched-chain production. However, branched-chains are produced minimally in gram-negative hosts (common microbial chassis).^51^ To validate the potential of this study, we propose a secondary approach for maximizing the enzymatic production of (S)-methylmalonyl-CoA and thus our branched-chains of interest. (S)-methylmalonyl-CoA can be secondarily produced by a side reaction of Acetyl-CoA Carboxylase (ACC). ACC normally reacts acetyl-CoA with HCO_3_^−^ to produce malonyl-CoA; however, occasionally propionyl-CoA is used in place of acetyl-CoA, generating (S)-methyl-malonyl-CoA. Under normal circumstances the cell degrades (S)-methylmalonyl-CoA back to propionyl-CoA by Methylmalonyl-CoA Decarboxylase (MMCD).^3^^,^^52^ Downregulation of MMCD leads to the accumulation of (S)-methylmalonyl-[acp], and thus the production of branched-chain fatty acids.^3^

### Electron uptake by H_2_-oxidation produces 2% higher energy conversion efficiencies than EEU

As seen in previous studies, H_2_-oxidation is a more efficient method for electron delivery than EEU.^8^^,^^25^^,^^26^^,^^34^ For single- and multi-methylated branched-chain hydrocarbons, use of H_2_-oxidation raises the electricity-to-fuel energy conversion efficiency by ≈ 2.3%. The energy-cost-savings of H_2_-mediated EMP over EEU-mediated EMP of single-branched-chain hydrocarbons ranges from 567 kJ mol^−1^ for 3-methyl-pentane (3-M_1_-pentane) to 1,000 kJ mol^−1^ for 5-methyl-decane (5-M_1_-decane). For multi-methylated branched-chain hydrocarbons, the energy savings range from 886 kJ mol^−1^ for 3,5-dimethyl-octane (3,5-M_2_-octane) to 1,250 kJ mol^−1^ for 2,4,6,8-tetramethyl-decane (2,4,6,8-M_4_-decane).

### Electromicrobial production could achieve synthesis of single-branched-chain alkanes at efficiencies between 34.7% and 40.0%

The energy requirements for synthesis of single-branched-chain alkanes range from ${11,000}_{-1,450}^{+193}\;\text{kJ}\;{\text{mol}}^{-1}$ for H_2_-driven 3-M_1_-pentane production to ${20,100}_{-2,710}^{+915}\;\text{kJ}\;{\text{mol}}^{-1}$ for EEU-driven 5-M_1_-decane (Figure 4A). The electrical-to-fuel energy conversion efficiencies of single-branched-chain alkane production range from $34.7_{-3.8}^{+1.7}\%$ for 2-M_1_-pentane with EEU, to $40.0_{-4.4}^{+0.7}\%$ for 2-M_1_-octane with H_2_ (Figure 4B). All of these efficiencies are close to the production efficiency for butanol (44% for the H_2_-driven Calvin cycle^8^) and glucose (44.6% for the H_2_-driven Calvin cycle^25^).

### Electromicrobial production could achieve synthesis of multi-branch-chain alkanes with efficiencies between 35.6% and 39.9%

The energy costs of EMP of multi-branched-chain alkanes range from ${14,000}_{-1,780}^{+238}\;\text{kJ}\;{\text{mol}}^{-1}$ for H_2_-derived 2,4-M_2_-hexane to ${25,400}_{-3,480}^{+1,150}\;\text{kJ}\;{\text{mol}}^{-1}$ for 2,4,6,8-M_4_-decane produced with EEU (Figure 4C). The electrical-to-fuel energy conversion efficiencies for multi-branched-chain alkanes are similar to those for single-branched-chain alkanes, ranging from $35.6_{-4.1}^{+1.7}\%$ for 2,5-M_2_-heptane with EEU to $39.9_{-4.54}^{+.7}\%$ for 2,4-M_2_-decane with H_2_ (Figure 4D).

### Increasing the fraction of branched-chain hydrocarbons in a jet fuel blend by 10% lowers conversion efficiency by 0.1%

We next calculated how the introduction of branched-chain alkanes into a jet fuel blend would change the energy production costs and energy conversion efficiency (Figure 5). We compared a previously conceived blend containing 85% straight-chain alkanes (C_10_-C_16_) and 15% terpenoids (pinene, limonene, farnesene, bisabolene, and geraniol),^34^ with two additional blends incorporating our branched-chain hydrocarbons (C_8_-C_10_ backbone). The energy conversion efficiency for H_2_-driven production of the original blend is $40.1_{-4.5}^{+0.7}\%$.

The first new blend contains 7% branched-chain alkanes, a minimum-allowed 8% terpenoids,^53^ and is filled out with 85% C_10_ to C_16_ straight-chain alkanes. The conversion efficiency for H_2_-driven production of this blend is slightly decreased to $40.0_{-4.6}^{+0.7}\%$. The energy conversion efficiency for H_2_-driven production 17% branched-chain alkanes, 8% terpenoid, and 75% straight-chain alkanes blend is further decreased to $39.9_{-4.6}^{+0.7}\%$. As we further increase branched-chain content, we see a continuation of this trend, with a drop in energy conversion efficiency of 0.1% for every additional 10% of branched-chain content (Figure 5).

## Discussion

Herein, we calculate conversion efficiencies of a panel of 13 single-branched and 11 multi-branched hydrocarbons with backbone lengths between C_5_ and C_10_, using H_2_-oxidation or EEU for electron delivery. Figures 4A and 4C show the energy required to produce a mole of each hydrocarbon, and Figures 4B and 4D show the electrical- and solar-to-chemical conversion efficiency for each molecule. Figure 5 shows the energy costs and production efficiencies of jet fuel blends containing increasing amounts of branched-chain alkanes.

We observe a general trend of increased energy cost with increased chain length. However, changing the position of the methylation sites can change the energy cost of synthesis. For example, the production of 3-M_1_-hexane is more expensive than 2-M_1_-hexane (see Figure 2C). This difference in energy is due to the higher energy cost for production of the (S)-methyl-malonyl-[acp] lengthener needed to install the branch in 3-M_1_-hexane, versus the cost of the 2-methyl-propionyl-[acp] initiator needed to install the branch in 2-M_1_-hexane. The high cost of (S)-methyl-malonyl-[acp], which adds 2 carbons to backbone, is due to the high energy cost of synthesis, requiring 15 ATP and 7 NADH (7.5 ATP C^−1^ and 3.5 NADH C^−1^). In contrast, 2-methyl-propionyl-[acp] adds 3 carbons to the backbone and requires 14 ATP and 10 NADH (4.6 ATP C^−1^ and 3.3 NADH C^−1^).

The production efficiency of odd-length alkanes with a branch on the second carbon is lower than that for even-length alkanes with the branch in the same place. Odd-alkanes with a branch on the second carbon need to be initiated with energy-expensive 3-methyl-butanoyl-[acp]. On the other hand, even-length alkanes with the branch in the same place need to be initiated with energy-cheap 2-methyl-propionyl-[acp] (see 2-M_1_-pentane and 2-M_1_-hexane an example in Figure 2C). Synthesis of 2-methyl-propionyl-[acp] in total costs 14 ATP and 10 NADH and adds 3 carbons to backbone (4.6 ATP C^−1^ and 3.3 NADH C^−1^). In contrast, 3-methyl-butanoyl-[acp] adds 4 carbons to the backbone, but requires 21 ATP and 14 NADH (5.25 ATP C^−1^ and 3.5 NADH C^−1^).

The efficiency of production of straight-chain alkanes ranges from $35.8_{-4.2}^{+1.7}\%$ for hexane to $40.8_{-4.5}^{+0.7}\%$ for hexadecane.^34^ Though similar to the efficiencies of our branched-chains, our straight-chains appear to have slightly higher efficiencies across the board, likely a result of higher combustion energies for compounds of similar carbon length. In all cases, if the electricity for production of these alkanes is derived from a perfectly efficient solar photovoltaic,^47^ then their production efficiency exceeds the efficiency of all forms of photosynthesis (see the right hand axis in Figure 4B).

As with single-branched-chain alkanes there is a general increase in energy requirement with multi-branched-chain hydrocarbon length. Furthermore, as with single-branched-chain alkanes, the choice of initiator molecule and the sites of branching cause notable differences in energy cost. The drop of energy required for 3,5-dimethyl octane over 2,4-dimethyl octane can be attributed to this cause. Here, 3,5-dimethyl octane obtains its branches entirely from the use of (S)-methylmalonyl-[acp], which is energetically unfavorable given its use of propionyl-[acp] in production. In contrast, 2,4-M_2_-octane requires the initial production of 2-methyl-propionyl-[acp], and further utilization of (S)-methylmalonyl-[acp]. 2-methyl-propionyl-[acp] is less energetically expensive to produce and thus costs less than (S)-methylmalonyl-[acp] alone. Therefore, though both molecules are chemically similar and combust similarly, they differ in production cost by ∼ $600\;\text{kJ}\;{\text{mol}}^{-1}$.

### Limitations of the study

We can already foresee significant challenges on the way to achieving the solar-to-fuel and electrical-to-fuel efficiencies predicted in this article. These challenges, and their effect on efficiency, are hard to predict but could come from space-time yields, kinetics and other process parameters not considered here. In an earlier work,^8^ we noted the effect of H_2_ solubility and of biofilm conductivity on the kinetics of the EMP-process and their subsequent effect on efficiency.^8^ If CO_2_-concentration is required for the EMP process, this could result in an additional efficiency cost (especially in the future when point CO_2_ sources like coal- and natural gas-fired power plants will hopefully be retired).^54^^,^^55^

However, we do not believe the challenges of realizing something close to our predicted efficiencies are insurmountable. For example, in 1961, Shockley and Quiesser made their estimate of the upper limit of the efficiency of solar photovoltaics when the highest reported efficiency of a PV device was ≈4%.^56^ Today, just a little over 60 years later, the technology is beginning to reach full maturity with efficiencies approaching 30%^57^ (the theoretical maximum is 33% ^58^), and costs that are exponentially reducing (Swanson’s law^59^). We can similarly envision that the cost (and perhaps the efficiency) of CO_2_-concentration technologies will drop rapidly due to learning-by-doing.^60^ While we may not know how to solve all of the engineering challenges of EMP, this article indicates that if they can be solved, the payoff could be significant. This article also allows the reader to separate which engineering interventions will have a big pay-off from those that will not. We believe this will be a particularly strong source of motivation and reassurance for young scientists working in the field.

First, as we noted in the results, we assume that the very low whole-cell voltages achieved at lab-scale can be consistently achieved when scaled-up. It is likely that this may not be initially feasible due to mass transport considerations. Likewise, limitations of carbon-fixing metabolism may also limit the achievable efficiency due to photorespiration and reduce efficiency by ≈ 25%.^48^

A very low whole-cell voltage could be achieved (perhaps even at large scale) using high salinity electrolytes. This would necessitate the use of an EMP organism that could tolerate high salinity. While most organisms used in EMP (especially those operated by EEU) are poorly tolerant of salinity, the highly engineerable *Vibrio natriegens* was recently discovered to be EEU-capable and is well known for being halophilic.^61^ The high natural tolerance to salinity and high evolvability of *V. natriegens* creates the possibility of operating an EMP system at very low whole-cell voltages and high efficiencies. These losses could also be reduced by creative reactor design as well.

Next, use of the Calvin cycle (as we consider in this article) is likely to cause efficiency losses due to photorespiration.^48^ Again, however, creative engineering could reduce these losses. Operating RuBisCO inside of a carbon concentrating mechanism like a carboxysome^62^ or bacterial nanocompartment^63^ could significantly reduce the oxygenation activity of RuBisCO, allowing the system efficiency to operate much closer to our theoretical maximum efficiency. Furthermore, swapping the entire carbon fixation cycle could eliminate oxygenation entirely. For instance, the 3HP-4HB cycle^17^ relies upon the Phosphoenol Pyruvate (PEP) carboxylase that does not suffer from the same oxygenation side reaction as RuBisCO, again allowing a much higher theoretical efficiency to be achieved. If a compartmentalization system can be implemented that completely shields enzymes from O_2_, then we could operate O_2_-sensitive pathways like the Wood-Ljungdahl pathway that could achieve very high efficiencies.^4^^,^^8^^,^^25^ For example, in our earlier work on the synthesis of blends of straight-chain alkanes and terpenoids, swapping the Calvin cycle for the Wood-Ljngdahl pathway raised the energy conversion efficiency of a jet fuel blend from 40.1% to 49.2%.^4^

Rate, yield, and titer are important concerns for EMP systems.^64^ For the production of branched-chain alkanes, many lessons can be learned from gram-negative bacteria that accumulate branched-chain lipids in their cell membranes, such as *B. subtilis*.^65^

Making this work a reality will require extensive metabolic engineering and synthetic biology, concerning both the creation of novel and potentially toxic pathways for producing hydrocarbons in combination with the engineering of radical new hosts for EMP. However, we have established that the high theoretical efficiency of EMP justifies doing this engineering in the hopes of creating viable, sustainable biofuels for demanding applications like aviation. Further, by using EMP to create a library of diverse, branched hydrocarbons that go beyond simple unbranched alkanes, we can create a repository of fuel components which when blended can replicate the desirable attribute of today’s fuels, furthering the cause of biofuels ultimately sourced from renewable electricity and CO_2_.

## STAR★Methods

### Key resources table

**Table:** 

REAGENT or RESOURCE	SOURCE	IDENTIFIER
**Software and algorithms**		

Python 3.9.6	Python Software Foundation	https://www.python.org
iPython 7.2.6.0	The iPython Development Team	https://www.ipython.org
Model code	This publication	https://github.com/barstowlab/emp-to-branched-jet

### Resource availability

#### Lead contact

Further information and requests for resources and reagents should be directed to and will be fulfilled by the lead contact, Buz Barstow (bmb35@cornell.edu).

#### Materials availability

This study did not generate any unique reagents.

#### Data and code availability

• Relevant data has been deposited at Zenodo^44^ and are publicly available as of the date of publication. DOIs are listed in the key resources table.
• All original code has been deposited at Github and archived at Zenodo^44^ and is publicly available as of the date of publication. DOIs are listed in the key resources table.
• Any additional information required to reanalyze the data reported in this paper is available from the lead contact upon request.

### Method details

Reaction tables for matrix generation and reduction were gathered from Kyoto Encyclopedia of Genes and Genomes (KEGG)^41^^,^^42^^,^^43^ and are summarized in Table 2. All equations utilized to arrive at energy costs and efficiency results are elaborated on in the **Results** and applied values can be found in Table 1. Computer models of this system were performed with iPython version 7.26.0 with Python 3.9.6 (for details, see key resources table). Graphs were produced with DataGraph, and graphics were produced with Adobe Illustrator.

## References

[1] Schnepf R., Yacobucci B.D. (2013).

[2] Hannon M., Gimpel J., Tran M., Rasala B., Mayfield S. (2010). Biofuels from algae: challenges and potential. Biofuels.

[3] Dewulf J.P., Gerin I., Rider M.H., Veiga-da-Cunha M., Van Schaftingen E., Bommer G.T. (2019). The synthesis of branched-chain fatty acids is limited by enzymatic decarboxylation of ethyl- and methylmalonyl-CoA. Biochem. J..

[4] Sheppard T.J., Specht D.A., Barstow B. (2023). Upper limit efficiency estimates for electromicrobial production of drop-in jet fuels. Bioelectrochemistry.

[5] Hellier P., Al-Haj L., Talibi M., Purton S., Ladommatos N. (2013). Combustion and emissions characterization of terpenes with a view to their biological production in cyanobacteria. Fuel.

[6] Mascal M., Dutta S. (2020). Synthesis of highly-branched alkanes for renewable gasoline. Fuel Process. Technol..

[7] Claassens N.J., Cotton C.A.R., Kopljar D., Bar-Even A. (2019). Making quantitative sense of electromicrobial production. Nat. Catal..

[8] Salimijazi F., Kim J., Schmitz A.M., Grenville R., Bocarsly A., Barstow B. (2020). Constraints on the Efficiency of Engineered Electromicrobial Production. Joule.

[9] Salimijazi F., Parra E., Barstow B. (2019). Electrical Energy Storage with Engineered Biological Systems. J. Biol. Eng..

[10] Rabaey K., Rozendal R.A. (2010). Microbial electrosynthesis - Revisiting the electrical route for microbial production. Nat. Rev. Microbiol..

[11] Rabaey K., Girguis P., Nielsen L.K. (2011). Metabolic and practical considerations on microbial electrosynthesis. Curr Opin Biotech.

[12] Lips D., Schuurmans J.M., Branco Dos Santos F., Hellingwerf K.J. (2018). Many ways towards ‘solar fuel’: Quantitative analysis of the most promising strategies and the main challenges during scale-up. Energy Environ. Sci..

[13] Prévoteau A., Carvajal-Arroyo J.M., Ganigué R., Rabaey K. (2020). Microbial electrosynthesis from CO2: forever a promise?. Curr Opin Biotech.

[14] Kim S., Lindner S.N., Aslan S., Yishai O., Wenk S., Schann K., Bar-Even A. (2020). Growth of E. coli on formate and methanol via the reductive glycine pathway. Nat. Chem. Biol..

[15] Abel A.J., Hilzinger J.M., Arkin A.P., Clark D.S. (2020). Systems-informed genome mining for electroautotrophic microbial production. bioRxiv.

[16] Hann E.C., Overa S., Harland-Dunaway M., Narvaez A.F., Le D.N., Orozco-Cárdenas M.L., Jiao F., Jinkerson R.E. (2022). A hybrid inorganic–biological artificial photosynthesis system for energy-efficient food production. Nat. Food.

[17] Claassens N.J., Sousa D.Z., dos Santos V.A.P.M., de Vos W.M., van der Oost J. (2016). Harnessing the power of microbial autotrophy. Nat. Rev. Microbiol..

[18] Torella J.P., Gagliardi C.J., Chen J.S., Bediako D.K., Colón B., Way J.C., Silver P.A., Nocera D.G. (2015). Efficient solar-to-fuels production from a hybrid microbial-water-splitting catalyst system. Proc. Natl. Acad. Sci. USA.

[19] Liu C., Colón B.C., Ziesack M., Silver P.A., Nocera D.G. (2016). Water splitting biosynthetic system with CO_2_ reduction efficiencies exceeding photosynthesis. Science.

[20] Guan X., Erşan S., Hu X., Atallah T.L., Xie Y., Lu S., Cao B., Sun J., Wu K., Huang Y. (2022). Maximizing light-driven CO(2) and N(2) fixation efficiency in quantum dot-bacteria hybrids. Nat. Catal..

[21] Rowe A.R., Salimijazi F., Trutschel L., Sackett J., Adesina O., Anzai I., Kugelmass L.H., Baym M.H., Barstow B. (2021). Identification of a Pathway for Electron Uptake in *Shewanella oneidensis*. Commun. Biol..

[22] Bai W., Ranaivoarisoa T.O., Singh R., Rengasamy K., Bose A. (2021). n-Butanol production by Rhodopseudomonas palustris TIE-1. Commun. Biol..

[23] Haas T., Krause R., Weber R., Demler M., Schmid G. (2018). Technical photosynthesis involving CO2 electrolysis and fermentation. Nat. Catal..

[24] Leger D., Matassa S., Noor E., Shepon A., Milo R., Bar-Even A. (2021). Photovoltaic-driven microbial protein production can use land and sunlight more efficiently than conventional crops. Proc. Natl. Acad. Sci. USA.

[25] Wise L., Marecos S., Randolph K., Hassan M., Nshimyumukiza E., Strouse J., Salimijazi F., Barstow B. (2022). Thermodynamic Constraints on Electromicrobial Protein Production. Front. Bioeng. Biotechnol..

[26] Marecos S., Brigham R., Dressel A., Gaul L., Li L., Satish K., Tjokorda I., Zheng J., Schmitz A.M., Barstow B. (2022). Practical and thermodynamic constraints on electromicrobially accelerated CO2 mineralization. iScience.

[27] Jeswani H.K., Chilvers A., Azapagic A. (2020). Environmental sustainability of biofuels: a review. Proc. Math. Phys. Eng. Sci..

[28] Brenner M.P., Bildsten L., Dyson F., Fortson N., Garwin R., Grober R., Hemley R., Hwa T., Joyce G., Katz J., Koonin S. (2006). https://apps.dtic.mil/docs/citations/ADA457082.

[29] Peralta-Yahya P.P., Zhang F., del Cardayre S.B., Keasling J.D. (2012). Microbial engineering for the production of advanced biofuels. Nature.

[30] Bai W., Geng W., Wang S., Zhang F. (2019). Biosynthesis, regulation, and engineering of microbially produced branched biofuels. Biotechnol. Biofuels.

[31] Sorigué D., Légeret B., Cuiné S., Blangy S., Moulin S., Billon E., Richaud P., Brugière S., Couté Y., Nurizzo D. (2017). An algal photoenzyme converts fatty acids to hydrocarbons. Science.

[32] Yunus I.S., Wichmann J., Wördenweber R., Lauersen K.J., Kruse O., Jones P.R. (2018). Synthetic metabolic pathways for photobiological conversion of CO2 into hydrocarbon fuel. Metab. Eng..

[33] Adesina O., Anzai I.A., Avalos J.L., Barstow B. (2017). Embracing Biological Solutions to the Sustainable Energy Challenge. Chem.

[34] Sheppard T.J., Specht D., Barstow B. (2022). Upper Limit Efficiency Estimates for Electromicrobial Production of Drop-In Jet Fuels. bioRxiv.

[35] Bird L.J., Bonnefoy V., Newman D.K. (2011). Bioenergetic challenges of microbial iron metabolisms. Trends Microbiol..

[36] Firer-Sherwood M., Pulcu G.S., Elliott S.J. (2008). Electrochemical interrogations of the Mtr cytochromes from Shewanella: Opening a potential window. J. Biol. Inorg. Chem..

[37] Ueki T., Nevin K.P., Woodard T.L., Aklujkar M.A., Holmes D.E., Lovley D.R. (2018). Construction of a Geobacter Strain With Exceptional Growth on Cathodes. Front. Microbiol..

[38] Kliphuis A.M.J., Klok A.J., Martens D.E., Lamers P.P., Janssen M., Wijffels R.H. (2012). Metabolic modeling of Chlamydomonas reinhardtii: energy requirements for photoautotrophic growth and maintenance. J. Appl. Phycol..

[39] Rowe A.R., Rajeev P., Jain A., Pirbadian S., Okamoto A., Gralnick J.A., El-Naggar M.Y., Nealson K.H. (2018). Tracking electron uptake from a cathode into *Shewanella* cells: Implications for energy acquisition from solid-substrate electron donors. mBio.

[40] Jiang W., Jiang Y., Bentley G.J., Liu D., Xiao Y., Zhang F. (2015). Enhanced production of branched-chain fatty acids by replacing beta-ketoacyl-(acyl-carrier-protein) synthase III (FabH). Biotechnol. Bioeng..

[41] Kanehisa M., Goto S. (2000). KEGG: Kyoto Encyclopedia of Genes and Genomes. Nucleic Acids Res..

[42] Kanehisa M. (2019). Toward understanding the origin and evolution of cellular organisms. Protein Sci..

[43] Kanehisa M., Furumichi M., Sato Y., Ishiguro-Watanabe M., Tanabe M. (2021). KEGG: integrating viruses and cellular organisms. Nucleic Acids Res..

[44] Sheppard T.J. (2023). Release of EMP-to-Branched-Jet for Preprint. Zenodo.

[45] Milo R., Jorgensen P., Moran U., Weber G., Springer M. (2010). BioNumbers - the database of key numbers in molecular and cell biology. Nucleic Acids Res..

[46] SunShot 2030 (2016). US Department of Energy. https://www.energy.gov/eere/solar/articles/2030-solar-cost-targets.

[47] Nelson J. (2003).

[48] Zhu X.-G., Long S.P., Ort D.R. (2008). What is the maximum efficiency with which photosynthesis can convert solar energy into biomass?. Curr. Opin. Biotechnol..

[49] Zhu X.-G., Long S.P., Ort D.R. (2010). Improving Photosynthetic Efficiency for Greater Yield. Annu. Rev. Plant Biol..

[50] Wijffels R.H., Barbosa M.J. (2010). An Outlook on Microalgal Biofuels. Science.

[51] Bentley G.J., Jiang W., Guamán L.P., Xiao Y., Zhang F. (2016). Engineering Escherichia coli to produce branched-chain fatty acids in high percentages. Metab. Eng..

[52] Linster C.L., Noël G., Stroobant V., Vertommen D., Vincent M.F., Bommer G.T., Veiga-da-Cunha M., Van Schaftingen E. (2011). Ethylmalonyl-CoA decarboxylase, a new enzyme involved in metabolite proofreading. J. Biol. Chem..

[53] Graham J.L., Striebich R.C., Myers K.J., Minus D.K., Harrison W.E. (2006). Swelling of nitrile rubber by selected aromatics blended in a synthetic jet fuel. Energy Fuels.

[54] Hack J., Maeda N., Meier D.M. (2022). Review on CO2 capture using amine-functionalized materials. ACS Omega.

[55] Pellegrini L.A., De Guido G., Consonni S., Bortoluzzi G., Gatti M. (2015). From biogas to biomethane: how the biogas source influences the purification costs. Chem. Eng. Trans..

[56] Gertner J. (2012).

[57] Green M.A., Dunlop E.D., Yoshita M., Kopidakis N., Bothe K., Siefer G., Hao X. (2023). Solar cell efficiency tables (version 62). Prog. Photovoltaics.

[58] Shockley W., Queisser H.J. (1961). Detailed Balance Limit of Efficiency of p-n Junction Solar Cells. J. Appl. Phys..

[59] Carr G. (2012). Sunny Uplands: Alternative energy will no longer be alternative. Economist.

[60] Lackner K.S., Azarabadi H. (2021). Buying down the Cost of Direct Air Capture. Ind. Eng. Chem. Res..

[61] Lam B.R., Barr C.R., Rowe A.R., Nealson K.H. (2019). Differences in Applied Redox Potential on Cathodes Enrich for Diverse Electrochemically Active Microbial Isolates From a Marine Sediment. Front. Microbiol..

[62] Flamholz A.I., Dugan E., Blikstad C., Gleizer S., Ben-Nissan R., Amram S., Antonovsky N., Ravishankar S., Noor E., Bar-Even A. (2020). Functional reconstitution of a bacterial CO_2_ concentrating mechanism in *E. coli*. Elife.

[63] Kirst H., Ferlez B.H., Lindner S.N., Cotton C.A.R., Bar-Even A., Kerfeld C.A. (2022). Toward a glycyl radical enzyme containing synthetic bacterial microcompartment to produce pyruvate from formate and acetate. Proc. Natl. Acad. Sci. USA.

[64] Keasling J., Garcia Martin H., Lee T.S., Mukhopadhyay A., Singer S.W., Sundstrom E. (2021). Microbial production of advanced biofuels. Nat. Rev. Microbiol..

[65] Kaneda T. (1991). Iso-and anteiso-fatty acids in bacteria: biosynthesis, function, and taxonomic significance. Microbiol. Rev..

