# Supplementary material for: Efficiency estimates for electromicrobial production of branched-chain hydrocarbons
Source: iScience. 2023 Dec 21;27(1):108773. doi: 10.1016/j.isci.2023.108773 (PMC10821168; doi:10.1016/j.isci.2023.108773)
Supplement: Document S1. Tables S1–S3 [file mmc1.pdf]

iScience, Volume *27*

Supplemental information

Efficiency estimates for electromicrobial production of branched-chain hydrocarbons

Timothy J. Sheppard, David A. Specht, and Buz Barstow

Supplementary Information for:

Efficiency Estimates for Electromicrobial Production of Branched-chain Hydrocarbons

Timothy J. Sheppard[1], David Specht[1], and Buz Barstow[1†]

[1]Department of Biological and Environmental Engineering, Cornell University, Ithaca, NY 14853, USA

†Corresponding author:

Buz Barstow, 228 Riley-Robb Hall, Cornell University, Ithaca, NY 14853; bmb35@cornell.edu

Supplementary Information Tables

Table S1. Symbols used in this article.

Table S2. NAD(P)H, reduced Ferredoxin, and ATP needed for synthesis of branched-chain alkanes.

Table S3. Molecular weights and energy densities for branched-chain alkanes.

**Symbol**	**Unit**	**Description**
E_{HC}	J molecule^{-1}	Energy carried per hydrocarbon molecule.
$\dot{N}_{HC}$	molecule s^{-1}	Hydrocarbon molecules produced per second.
N_A	molecule mol^{-1}	Avogadro constant.
$P_{e, T}$	J s^{-1}	Total electrical power input into electromicrobial production system.
L_{EP}	kJ mol^{-1}	Electrical energy cost to generate one mole of product.
C_{SP}	¢ mol^{-1}	Minimum solar electricity cost for synthesis of one mole of product at 2030 solar electricity
M_{HC}	g mol^{-1}	Molecular weight of hydrocarbon molecule.
η_{EP}	%	Electrical to product (*e.g.,* hydrocarbon) energy conversion efficiency.
η_{SP}	%	Solar to product (*e.g.,* hydrocarbon) energy conversion efficiency.
e	A s	Fundamental charge.
ν_{ep}	#	Number of electrons needed for synthesis of a product (*e.g.*, hydrocarbon) molecule.
$\Delta U_{e.cell}$	V	Potential difference across bio-electrochemical cell.
ξ_{I2}	#	Faradaic efficiency of the bio-electrochemical cell.
$\nu_{p, NADH}$	#	Number of NAD(P)H molecules needed to make a final product molecule.
$\nu_{p, Fd}$	#	Number of ferredoxin molecules needed to make a final product molecule.
$\nu_{p, ATP}$	#	Number of ATP molecules needed to make a final product molecule.
$\Delta G_{ATP/ADP}$	J	Free energy for regeneration of ATP.
$\Delta U_{membrane}$	V	Inner membrane potential difference.
U_{H2}	V	Standard potential of proton reduction to H_2.
$U_{acceptor}$	V	Standard potential of terminal electron acceptor reduction.
U_Q	V	Redox potential of the inner membrane electron carrier.
U_{NADH}	V	Standard potential of NADH.
U_{Fd}	V	Standard potential of Ferredoxin.

Table S1. Symbols used in this article.

Branched-chain Alkane	No. ATP	No. NAD(P)H	No. Reduced Ferredoxin	No. CO_2
2-M_1-pentane	29	22	0	6
2-M_1-hexane	31	24	0	7
2-M_1-heptane	37	28	0	8
2-M_1-octane	39	30	0	9
3-M_1-pentane	30	21	0	6
3-M_1-hexane	37	24	0	7
3-M_1-heptane	38	27	0	8
3-M_1-octane	45	30	0	9
3-M_1-nonane	46	33	0	10
4-M_1-octane	45	30	0	8
4-M_1-decane	53	36	0	11
5-M_1-nonane	46	33	0	10
5-M_1-decane	53	36	0	11
2,4-M_2-hexane	37	27	0	8
2,4-M_2-octane	46	33	0	10
2,4-M_2-decane	54	39	0	12
2,5-M_2-heptane	44	31	0	9
2,5-M_2-nonane	52	37	0	11
3,5-M_2-octane	552	33	0	10
3,5-M_2-decane	60	39	0	12
4,6-M_2-decane	60	39	0	12
2,4,6-M_3-octane	53	36	0	11
2,4,6-M_3-decane	61	42	0	13
2,4,6,8-M_4-decane	68	45	0	14

Table S2. NAD(P)H, reduced Ferredoxin, and ATP needed for synthesis of single molecules of branched-chain alkanes from CO_2. All alkanes are synthesized from CO_2 with the Calvin cycle, Type II Fatty Acid Synthesis and with the ADO decarboxylation pathway. Calculated with the INFO-FIG4A&B.PY, INFO-FIG4C&D.PY codes in the EMP-TO-BRANCHED-JET repository [Sheppard2023b].

Branched-chain Alkane	Molecular Weight (Da)	Energy Density (kJ mol^{-1})	Energy Density (J molecule^{-1})
2-M_1-pentane	86.18	4,153.80	6.90E-18
2-M_1-hexane	100.2	4,809.98	7.99E-18
2-M_1-heptane	114.23	5,466.16	9.08E-18
2-M_1-octane	128.26	6,140.40	1.02E-17
3-M_1-pentane	86.18	4,159.82	6.91E-18
3-M_1-hexane	100.2	4,816.00	8.00E-18
3-M_1-heptane	114.23	5,478.20	9.10E-18
3-M_1-octane	128.26	6,140.40	1.02E-17
3-M_1-nonane	142.28	6,742.40	1.12E-17
4-M_1-octane	128.26	6,140.40	1.02E-17
4-M_1-decane	156.31	7,404.60	1.23E-17
5-M_1-nonane	142.28	6,742.40	1.12E-17
5-M_1-decane	156.31	7,404.60	1.23E-17
2,4-M_2-hexane	114.23	5,461.69	9.07E-18
2,4-M_2-octane	142.28	6,768.13	1.12E-17
2,4-M_2-decane	170.33	8,074.58	1.34E-17
2,5-M_2-heptane	128.26	6,114.91	1.02E-17
2,5-M_2-nonane	156.31	7,421.35	1.23E-17
3,5-M_2-octane	142.28	6,768.13	1.12E-17
3,5-M_2-decane	170.33	8,074.58	1.34E-17
4,6-M_2-decane	170.33	8,074.58	1.34E-17
2,4,6-M_3-octane	156.31	7,421.35	1.23E-17
2,4,6-M_3-decane	184.36	8,727.80	1.45E-17
2,4,6,8-M_4-decane	198.39	9,381.02	1.56E-17

Table S3. Molecular weights and energy densities for branched-chain alkanes. Data from NIST database [NIST2022a].

Supplementary Information References

[NIST2022a] P.J. Linstrom and W.G. Mallard, Eds., NIST Chemistry WebBook, NIST Standard Reference Database Number 69, National Institute of Standards and Technology, Gaithersburg MD, 20899 (retrieved September 24, 2022). doi:10.18434/T4D303.

[Sheppard2023b] T.J. Sheppard and B. Barstow. EMP-to-Branched-Jet. Archived on Zenodo (2023). doi:10.5281/zenodo.7693794.
